# Antibody levels in a cohort of pregnant women after the 2009 influenza A(H1N1) pandemic: Waning and association with self‐reported severity and duration of illness

**DOI:** 10.1111/irv.12623

**Published:** 2019-01-01

**Authors:** Gro Tunheim, Ida Laake, Anna Hayman Robertson, Kristian Waalen, Olav Hungnes, Lisbeth M. Næss, Rebecca J. Cox, Siri Mjaaland, Lill Trogstad

**Affiliations:** ^1^ Division of Infection Control and Environmental Health Norwegian Institute of Public Health Oslo Norway; ^2^ K. G. Jebsen Centre for Influenza Vaccine Research University of Oslo Oslo Norway; ^3^ The Influenza Centre Department of Clinical Science University of Bergen Bergen Norway; ^4^ Department of Research and Development Haukeland University Hospital Bergen Norway

**Keywords:** antibodies, influenza, pandemic H1N1pdm09, pregnancy, vaccination, waning

## Abstract

**Background:**

A population‐based pregnancy cohort was established in Norway to study potential effects of exposure to the 2009 influenza pandemic or pandemic vaccination during pregnancy.

**Objectives:**

We studied maternal A(H1N1)pdm09‐specific hemagglutination inhibition (HI)‐titer levels and waning in women with influenza‐like illness (ILI) in pregnancy compared to vaccinated women. Moreover, we studied the association between HI‐titers and self‐reported severity and duration of ILI.

**Methods:**

HI‐titers against the pandemic virus were measured in maternal blood samples obtained at birth, 3‐9 months after exposure, and linked with information about pregnancy, influenza and vaccination from national registries and a cohort questionnaire.

**Results:**

Among 1821 pregnant women included, 43.7% were unvaccinated and 19.3% of these had ILI. HI‐titers were low (geometric mean titer (GMT) 11.3) in the unvaccinated women with ILI. Higher HI‐titers (GMT 37.8) were measured in the vaccinated women. Estimated HI‐titer waning was similar for vaccinated women and women with ILI. Most ILI episodes were moderate and lasted 3‐5 days. Women with ILI reporting specific influenza symptoms such as fever or cough had higher HI‐titers than women without these symptoms. Women who reported being “very ill” or illness duration of >5 days had higher HI‐titers than women reporting less severe illness or illness of shorter duration, respectively.

**Conclusions:**

Antibody waning was similar in vaccinated women and women with ILI. More severe ILI or longer duration of illness was associated with higher HI‐titers. Most unvaccinated pregnant women with ILI had low HI‐titers, probably due to moderate illness and HI‐titer waning between exposure and sampling.

## 1 INTRODUCTION

During the 2009 influenza A(H1N1) pandemic, pregnant women were at increased risk of hospitalization and death due to severe influenza infection.^1^, ^2^, ^3^ Following the first reports on this association, a population‐based cohort of pregnant women (The Norwegian Influenza Cohort Study, NorFlu) was established in Norway, to study the potential effects of maternal pandemic influenza and vaccination on the women and their children.

In Norway, the main pandemic period occurred between October 1, 2009, to December 31, 2009, peaking in early November.^4^, ^5^ A vaccination campaign with the AS03‐adjuvanted A(H1N1)pdm09 vaccine (Pandemrix) started 19th October. The vaccine was recommended to pregnant women in their second or third trimester and to groups at high risk of severe influenza.^6^ It was mandatory to report pandemic vaccination to the national immunisation register (SYSVAK).^4^, ^5^ Approximately 54% of pregnant Norwegian women were vaccinated.^4^

In contrast, among 46 000 pregnancies in Norway during the pandemic, only 516 cases of laboratory‐confirmed influenza infection were registered. Due to limited laboratory capacity during the pandemic, testing of patients with severe illness was prioritized. Furthermore, only 8.9% of women who gave birth in 2009 or 2010 were diagnosed with influenza by a physician.^4^ However, national influenza surveillance data indicated a clinical attack rate of approximately 30% in the Norwegian population.^5^

Data on laboratory‐confirmed influenza were limited also for women in the NorFlu pregnancy cohort, but antibodies against the A(H1N1)pdm09 virus, measured as hemagglutination inhibition (HI)‐titers, were measured in maternal blood samples taken at delivery, 3‐9 months after the pandemic period. As the 2009 A(H1N1)pdm09 virus was an antigenically novel virus^7^ and the frequency of pre‐existing antibodies to the virus was low in the Norwegian population prior to the pandemic,^8^ high HI‐titers might serve as a proxy for infection in unvaccinated individuals.

Antibodies induced by influenza infection or vaccination are known to wane over time,^9^, ^10^, ^11^, ^12^, ^13^ and HI‐titers have been suggested to decline faster after vaccination with pandemic vaccines than after infection.^14^, ^15^, ^16^ Studying antibody waning is important for understanding the longevity of the maternal antibodies. A small study from the 2009 pandemic found no significant difference in HI‐titer waning between vaccinated and infected pregnant women, although the HI‐titers declined at a slightly slower rate in the infected women.^17^ As the immune system is altered to tolerate the fetus, pregnancy may influence the immune response to pandemic influenza infection and vaccination.^18^ However, studies on influenza vaccination of pregnant women mostly indicate that their immune responses are comparable to those of non‐pregnant healthy individuals.^19^

Due to the long time interval between the pandemic exposure and blood sampling at delivery, we aimed to estimate HI‐titer waning in the unvaccinated pregnant women with ILI during the 2009 pandemic, and compare with the estimated waning in the vaccinated women. In addition, as HI‐titers have been reported to be positively associated with influenza symptoms and severity of illness,^20^, ^21^ we also studied whether HI‐titers were associated with self‐reported ILI symptoms, severity or duration of ILI, in this cohort of pregnant women.

## 2 MATERIALS AND METHODS

### 2.1 The NorFlu cohort

Pregnant women with their last menstrual date between June 1, 2009, and December 31, 2009, were eligible for recruitment. Women who attended the routine ultrasound around pregnancy week 18 (offered to all pregnant women in Norway) were recruited from four hospitals in the Oslo and Bergen areas. A total of 3201 women (60%) agreed to participate, and informed consent was obtained from all participants. The study was approved by the Regional Committees for Medical and Health Research Ethics South East (2009/2165).

Blood samples were collected from the women at delivery. The participants completed a questionnaire on influenza and vaccination during pregnancy. Each participant′s cohort data were linked to national health registers by their personal identification number. Information about the pregnancy and vaccination was collected from the Medical Birth Registry of Norway (MBRN)^22^ and the Norwegian Immunisation Registry (SYSVAK),^23^ respectively. Information about primary care influenza diagnoses or laboratory‐confirmed pandemic infection was obtained from the Directorate of Health's reimbursement database for primary care consultations (KUHR) and from the Norwegian Surveillance System for Communicable Diseases (MSIS), respectively.

### 2.2 Hemagglutination inhibition (HI) assay

Maternal serum samples were tested in HI assays with the pandemic vaccine virus NYMC X‐179A, in twofold dilutions starting at 1:10.^24^ The HI‐titer was defined as the reciprocal of the highest serum dilution that completely inhibited turkey red blood cell agglutination. The HI‐titers were normalized against the human international standard 09/194 (NIBSC, UK).^25^ Samples with an HI‐titer below the level of detection were assigned a titer of 5 for calculation purposes.

### 2.3 Pandemic vaccination status

Women registered with one dose of the pandemic vaccine in SYSVAK (date of vaccination registered), or self‐reported pandemic vaccination in the questionnaire (month and year of vaccination), were defined as vaccinated.

### 2.4 Influenza and ILI categories

Women were defined as having “medically attended influenza” if they were registered with either an influenza diagnosis (code R80 in the International Classification of Primary Care‐2^26^) in the KUHR database and/or with laboratory‐confirmed A(H1N1)pdm09 infection in MSIS during the main pandemic period. Women who solely self‐reported ILI during the pandemic period were defined as having “self‐reported ILI.” For self‐reported ILI, the women reported month and year of disease. Women with either medically attended influenza or self‐reported ILI were defined as having “ILI.”

### 2.5 Study population

**Figure 1:**
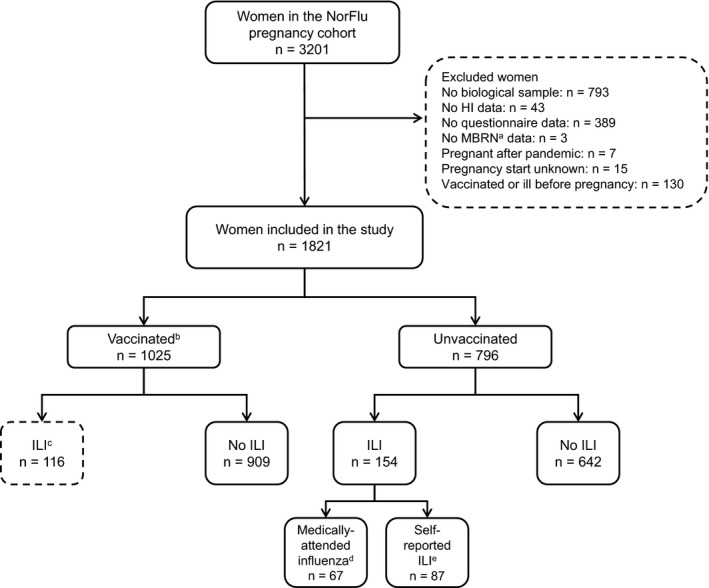
Overview of the study subjects. ^a^MBRN = The Medical Birth Registry containing information about all births in Norway. ^b^Vaccinated against A(H1N1)pdm09 with an AS03‐adjuvanted vaccine (Pandemrix, GSK). ^c^ILI = Influenza‐like illness: medically attended influenza or self‐report of ILI during the main pandemic period (Oct‐Dec 2009). ^d^Medically attended influenza: influenza diagnosis (code R80 in the International Classification of Primary Care‐2) and/or laboratory‐confirmed A(H1N1)pdm09 infection during the main pandemic period. ^e^Self‐reported ILI: ILI based on self‐report alone during the main pandemic period

Among the 3201 women in the cohort, participants were excluded if any of the following data were missing: biological samples, HI‐titer measurements, questionnaire data or MBRN records (Figure 1). Women were also excluded if their pregnancy started after the pandemic period, if their start‐date was unknown, or if they were not pregnant when they were vaccinated or had ILI. Women who self‐reported vaccination had to be pregnant by October 19, 2009, when the vaccination campaign started. For women with self‐reported ILI, month of illness could be no earlier than month of pregnancy start. Excluded women were similar to those who were included in terms of age, parity, and proportion registered in SYSVAK and with a primary care influenza diagnosis. However, for excluded women, the HI geometric mean titer (GMT) was slightly higher (19.9 vs 18.4), the mean interval between the peak pandemic and birth was longer (240 days vs 225 days) and fewer were registered in MSIS (0.6% vs 1.5%).

### 2.6 Time since exposure

Time since exposure was the interval in days from exposure (ILI or vaccination) to birth. If vaccination was not registered in SYSVAK (n = 111, 10.8% of vaccinations), the median date of vaccination in the cohort (November 9, 2009), was used. The dates 15th October, 1st November and 1st December 2009 were chosen as dates of exposure for illness in these months, based on the distribution of dates for the medically attended cases.

### 2.7 Symptoms, severity, and duration of ILI

Self‐reported symptoms were extracted from a list of common influenza symptoms in the questionnaire. “Fever > 39°C,” “fever < 39°C,” or “unmeasured fever” were combined to “fever” in the analysis. The US Centers for Disease Control and Prevention (CDC) case definition of ILI is fever >37.8°C, cough and/or a sore throat in the absence of another known cause.^27^ When classifying the women according to the CDC definition, the combined definition of “fever” described above was used instead of the criteria fever >37.8°C. Severity was based on the question “How ill did you feel?” with categories “not very ill,” “quite ill,” and “very ill.” Duration of ILI was based on the question “How long were you ill?” with categories “0‐2,” “3‐5,” or “more than 5 days.”

### 2.8 Statistical analysis

Characteristics of unvaccinated and vaccinated women were compared using a *t* test for continuous variables and a chi‐square test for categorical variables.

Linear regression analysis was used to estimate waning of HI‐titers among unvaccinated (n = 154) women with ILI and vaccinated women without ILI (n = 909).^17^ Women who had been both vaccinated and ill were excluded from the comparison. The log2‐transformed HI‐titers were regressed on time since exposure. The difference between the estimated rates of decline among women with ILI and vaccinated women was evaluated with an interaction term between time and type of exposure. Half‐life estimates were calculated from the rates of decline.^28^, ^29^

Preliminary analyses of the study population found no significant differences in HI‐titers between vaccinated women with or without ILI, possibly due to the high HI‐titers induced by the vaccine.^30^, ^31^ Analyses of HI‐titers in the different ILI categories were therefore restricted to unvaccinated women (n = 796). The association between HI‐titers and symptoms, severity and duration were studied in unvaccinated women with ILI (n = 154). For these analyses, we used a Wilcoxon signed rank‐sum test. Differences in proportions of women with HI‐titers <10 or ≥20 were compared using a chi‐square test. To ensure that any observed HI‐titer differences were not due to differences in time since exposure, subanalyses were performed for women with or without ILI giving birth ≥210‐≤280 days after the pandemic (>60% of the women) and for medically attended vs self‐report women giving birth ≥180‐≤270 days after the ILI episode (>70% of these women). A *P*‐value < 0.05 was considered statistically significant. Calculations were made using Stata/SE 14.0.

## 3 RESULTS

A total of 1821 pregnant women were included in the study. All babies were born between March and September 2010, and 88.3% of the questionnaires were completed either before birth or in the month of giving birth. Among the included women, 56.3% (n = 1025) were vaccinated with the pandemic vaccine. The majority of vaccinations (89.2%) were registered in SYSVAK (Table 1). HI‐titers were similar for women with self‐reported vaccination and SYSVAK registered vaccinations (data not shown). The pregnancy start‐date was earlier for vaccinated women, probably reflecting the national recommendation of vaccination during second and third trimester only. Parous women were more often vaccinated; however, the proportion of women with other risk factors for severe influenza illness was similar for vaccinated and unvaccinated women. The HI GMT at delivery was significantly higher in the vaccinated than in the unvaccinated women, while the number of days between the pandemic peak and delivery was significantly shorter in vaccinated compared to unvaccinated women (Table 1).

**Table 1:** Characteristics of included women

	All n = 1821	Vaccinated n = 1025 (56.3%)	Unvaccinated n = 796 (43.7%)	*P*‐values for differences between vaccinated and unvaccinated women^a^
Recorded in immunisation registry (SYSVAK), n (%)		914 (89.2)	0	‐
Age (y), mean (range)	31.9 (17‐45)	31.8 (17‐44)	32.0 (17‐45)	0.54
At least one previous birth, n (%)	896 (49.2)	527 (51.4)	369 (46.4)	**0.03**
Influenza risk factors^a^	154 (8.5)	91 (8.9)	63 (7.9)	0.46
Pregnancy start‐dates, median (range)	02 Sep 2009 (04 Jun 2009‐30 Dec 2009)	12 Aug 2009 (04 Jun 2009‐07 Dec 2009)	27 Sep 2009 (15 Jun 2009‐30 Dec 2009)	**<0.0001**
HI‐titer at delivery, GMT (95% CI)	18.4 (17.4, 19.5)	37.8 (35.4, 40.3)	7.3 (6.9, 7.7)	**<0.0001**
No of days from pandemic peak^b^ to date of birth, mean (range)	224 (140‐330)	208 (140‐319)	246 (143‐330)	**<0.0001**
Influenza diagnosis (R80) from primary health care, n (%)	105 (5.8)	47 (4.6)	58 (7.3)	**0.014**
Laboratory‐confirmed influenza, n (%)	28 (1.5)	1 (0.1)	27 (3.4)	**<0.001**
ILI^c^, n (%)	270 (14.8)	116 (11.3)	154 (19.3)	**<0.001**
Medically attended influenza^d^, n (%)	114 (6.3)	47 (4.6)	67 (8.4)	**0.001**
Self‐reported ILI^e^, n (%)	156 (8.6)	69 (6.7)	87 (10.9)	**0.001**
No ILI^f^, n (%)	1551(85.2)	909 (88.7)	642 (80.7)	**<0.001**

P‐values < 0.05 in bold.

Continuous variables tested with *t* test and categorical variables tested with Chi‐squared test.

a Asthma, diabetes 1 or 2, other lung disease, obesity, cardiovascular disease, kidney disease, impaired immune.

b Pandemic peak set to 2nd of Nov. 2009.

c ILI: Medically attended influenza or solely self‐reported ILI during the main pandemic period (Oct‐Dec 2009).

d Medically attended influenza: influenza diagnosis (code R80 in the International Classification of Primary Care‐2) and/or laboratory‐confirmed A(H1N1)pdm09 infection during the main pandemic period.

e Self‐reported ILI: ILI case based on self‐report alone during the main pandemic period.

f No ILI, i. e. no influenza‐like illness. Not registered with ILI during the main pandemic period.

Only 6.3% of the women had medically attended influenza during the pandemic. A total of 270 women (14.8%) had ILI; of these, 42.2% had medically attended influenza and 57.8% had self‐reported ILI (Table 1). Unvaccinated women had significantly more ILI than vaccinated women (19.3% vs 11.3%, respectively).

### 3.1 Estimation of HI‐titer waning

**Figure 2:**
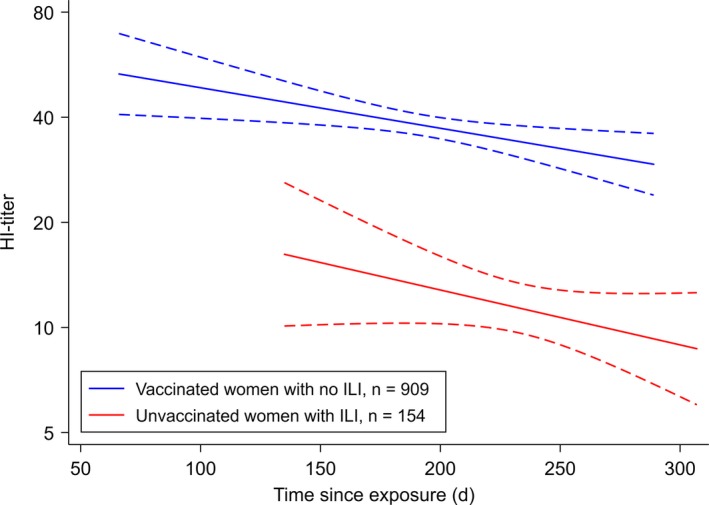
Estimated waning of HI‐titers in pregnant women after influenza‐like illness (ILI) or pandemic vaccination. The graph shows the log2‐transformed HI‐titers regressed on time since exposure with 95% confidence intervals (dashed curves). Time was defined as the interval in days from exposure (ILI or pandemic vaccination) to delivery

Figure 2 shows the estimated waning of HI‐titers over time since exposure for vaccinated women with no ILI (n = 909) and unvaccinated women with ILI (n = 154). The mean time since exposure was 194 days (range 66‐289) for vaccinated cases and 233 (range 135‐307) for ILI cases. HI‐titers were significantly higher in the vaccinated women than in women with ILI (*P* < 0.001). The HI‐titers declined significantly with time since exposure in the vaccinated women (*P* = 0.0009); however, >8 months after vaccination 50% of the women had HI‐titers ≥20. In women with ILI, waning was not significant (*P* = 0.11), probably due to the low number of women in this group. The estimated HI‐titer half‐life was 260 days for vaccinated women, and 192 days for women with ILI, suggesting a faster waning in women with ILI. However, the slopes of the two regression curves were not significantly different (*P* = 0.71).

Since medically attended influenza possibly represents cases more likely due to influenza virus and/or more severe illness than self‐reported ILI, a subanalysis was performed in unvaccinated women with medically attended influenza (n = 67). No significant HI‐titer waning was found (*P* = 0.49), and waning was not significantly different from the estimated waning in vaccinated women (*P* = 0.98). The estimated HI‐titer half‐life for the medically attended women was 250 days, slightly longer than for all women with ILI.

### 3.2 HI‐titers in the unvaccinated women

**Figure 3:**
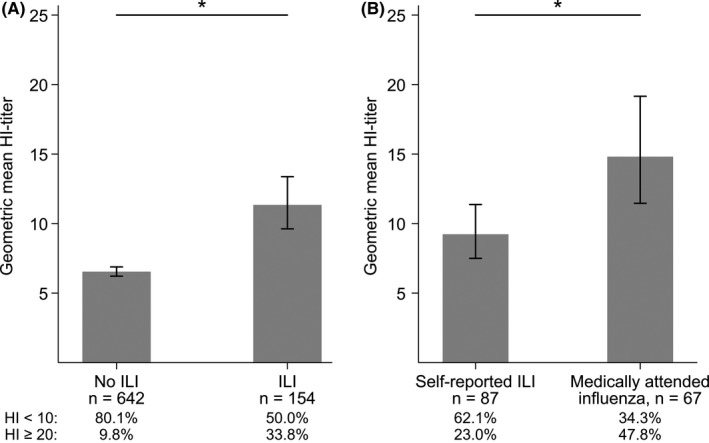
HI‐titers according to influenza‐like illness (ILI) status in unvaccinated, pregnant women after the 2009 pandemic (A) HI‐titers in women with no ILI or with ILI (B) HI‐titers in women with medically‐attended influenza and women who self‐reported ILI. The graphs show the geometric mean HI‐titers with 95% confidence intervals. *P ≤ 0.001 (Wilcoxon signed rank‐sum test)

We observed low HI‐titers (GMT 11.3) among the unvaccinated women with ILI; however, they had significantly higher HI‐titers than women with no ILI (GMT 6.5, *P* < 0.0001; Figure 3A). Several of the unvaccinated women with laboratory‐confirmed influenza also had low titers (11.1% with HI < 10 and 33.3% with HI < 20). 80.1% of the women with no ILI had HI < 10, compared to 50.0% of the women with ILI (*P* < 0.001). Conversely, 35% of women with ILI had HI‐titers ≥20 compared to 9.6% of women without ILI (*P* < 0.001). Women with medically attended influenza had significantly higher HI‐titers (*P* = 0.001, GMT 14.8) than women with self‐reported ILI (GMT 9.2; Figure 3B). Consistent with this, 47.8% of women with medically attended influenza had HI‐titers ≥20 compared to 23.0% of women with self‐reported ILI (*P* = 0.001). When analyzing subsets of women with similar time since exposure, the differences in HI‐titers were still significant between the previously mentioned groups (data not shown).

### 3.3 HI‐titers and self‐reported symptoms, severity and duration of ILI in unvaccinated women

**Figure 4:**
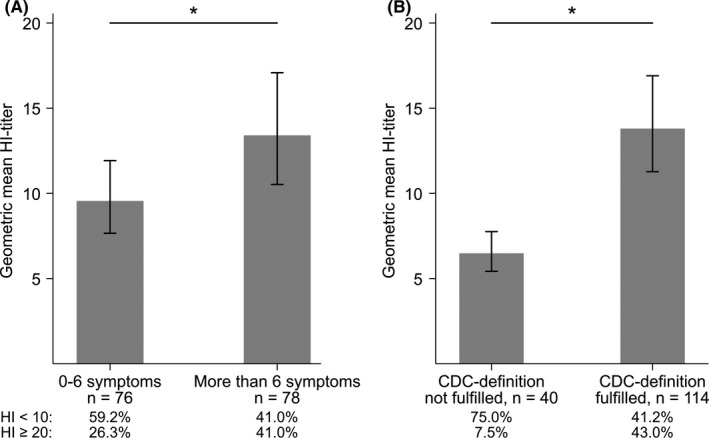
HI‐titers according to self‐reported symptoms in unvaccinated, pregnant women with influenza‐like illness (ILI) during the 2009 pandemic (A) HI‐titers in women reporting 0‐6 symptoms and more than six symptoms (B) HI‐titers in women with or without symptoms matching the CDC case definition of ILI. The graphs show the geometric mean HI‐titers with 95% confidence intervals. *P < 0.05 (Wilcoxon signed rank‐sum test)

The frequency of self‐reported symptoms in unvaccinated women with ILI is listed in Table 2. Women who reported fever, cough, sore throat, shortness of breath or chest pain had significantly higher HI‐titers than women without these specific symptoms (Table 2). The mean number of self‐reported symptoms was 5.8. Women who reported >6 symptoms had significantly higher HI‐titers (*P* = 0.02, GMT 13.4) than women with ≤6 symptoms (GMT 9.6; Figure 4A). ILI cases who matched the CDC definition of ILI^27^ (“CDC‐ILI”; 74.0%), had significantly higher HI‐titers (*P* < 0.0001, GMT 13.8) than the women who did not (GMT 6.5; Figure 4B). Similarly, 43.0% of the CDC‐ILI cases had HI‐titers ≥20 compared to 7.5% of the women not matching the definition, while 41.2% and 75.0%, respectively, had HI < 10 (*P* < 0.001).

**Table 2:** Frequency of self‐reported symptoms in unvaccinated women with influenza‐like illness (ILI; n = 153) and HI‐titers according to self‐report of individual symptoms

Women reporting the symptom, n (%)		Geometric mean HI‐titer (95% CI)		*P*‐values for differences in HI‐titer^a^
		If symptom reported	If symptom not reported	
Fever	128 (83.1)	12.6 (10.4, 15.1)	6.9 (5.3, 9.0)	**0.005**
Headache	117 (76.0)	11.7 (9.7, 14.2)	10.2 (7.2, 14.5)	0.31
Sore throat	114 (74.0)	13.0 (10.6, 15.9)	7.7 (6.0, 9.9)	**0.003**
Stuffy nose/runny nose	108 (70.1)	12.7 (10.3, 15.6)	8.7 (6.8, 11.2)	**0.06**
Cough	103 (66.9)	13.7 (11.1, 17.0)	7.7 (6.2, 9.6)	**0.0007**
Muscle pain	94 (61.0)	12.2 (9.9, 15.1)	10.1 (7.7, 13.2)	0.13
Joint pains	86 (55.8)	10.8 (8.8, 13.1)	12.1 (9.1, 16.1)	0.96
Shortness of breath	73 (47.4)	13.8 (10.6, 18.0)	9.5 (7.8, 11.6)	**0.04**
Vomiting, diarrhea	46 (29.9)	12.5 (9.3,16.9)	10.9 (8.9, 13.3)	0.30
Chest pain	21 (13.6)	17.5 (11.4, 27.0)	10.6 (8.9, 12.6)	**0.01**
Pneumonia	5 (3.3)	17.4 (3.7, 81.2)	11.2 (9.5, 13.2)	0.39

P‐values < 0.05 in bold.

a Wilcoxon rank‐sum test.

**Figure 5:**
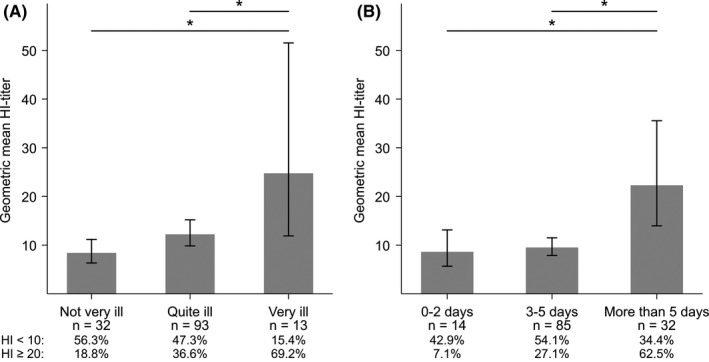
HI‐titers according to self‐reported severity and duration of influenza‐like illness (ILI) in unvaccinated, pregnant women with influenza‐like illness (ILI) during the 2009 pandemic (A) HI‐titers in women who were “not very ill,” “quite ill,” or “very ill” (B) HI‐titers in women who were ill for “0‐2,” “3‐5,” or “more than 5” days. The graphs show the geometric mean HI‐titers with 95% confidence intervals. *P < 0.05 (Wilcoxon signed rank‐sum test)

Most of the unvaccinated women with ILI felt “quite ill” (67.4%). Women who were “very ill” had significantly higher HI‐titers (GMT 24.8) compared to women who reported less severe ILI (either “not very ill” GMT 8.4, *P* = 0.002 or “quite ill” GMT 12.2, *P* = 0.03; Figure 5A). The proportion of women with an HI‐titer ≥20 increased from 18.8% to 69.2% with increasing severity (*P*‐values < 0.05 for “very ill” vs either “not very ill” or “quite ill”). Most women (64.9%) reported an ILI duration of 3‐5 days. HI‐titers were significantly higher in women who reported ILI for more than 5 days (GMT 22.3) than in women with a shorter duration of illness (GMT 8.6, *P* = 0.04 vs 0‐2 days and GMT 9.5, *P* = 0.001 vs 3‐5 days; Figure 5B). Again, there was a significantly higher proportion (*P* < 0.001) of women with HI‐titers ≥20 (62.5%) among women with more than 5 days of ILI than among women with shorter durations. A subanalysis of only the laboratory‐confirmed influenza cases (n = 27) was also performed. HI‐titers in these women showed similar associations with severity and duration as in all women with ILI, with the exception of women who reported to be “very ill” (n = 3 and data not shown). However, all subgroups were very small (range n = 3‐15).

## 4 DISCUSSION

In this unique cohort of women who were pregnant during the 2009 influenza A(H1N1) pandemic, HI‐titers against the pandemic virus were measured in maternal blood samples collected at delivery, 3‐9 months after exposure to pandemic infection or vaccination. We found that HI‐titers were higher after vaccination with the AS03‐adjuvanted vaccine than after ILI. HI‐titers waned over time, both for vaccinated women and for unvaccinated women with ILI. The estimated HI‐titer waning was comparable between these groups. HI‐titers were higher among women reporting either more severe ILI or longer duration of ILI, compared to women with less severe ILI or ILI of shorter duration, respectively.

Immune responses after pandemic vaccination or natural infection with influenza A(H1N1)pdm09 virus may be qualitatively and quantitatively different in non‐pregnant individuals.^32^ However, few studies have compared HI‐titers after pandemic vaccination and infection in pregnant women. In accordance with a Danish study, we found that the AS03‐adjuvanted pandemic vaccine had induced significantly higher HI‐titers than ILI in pregnant women.^30^ This finding is in contrast to a US study, where vaccination and infection induced similar HI‐titers in pregnant women; however, there a non‐adjuvanted vaccine was used, all influenza cases were medically attended, and time since exposure was shorter.^17^

Although we found that unvaccinated women with ILI had higher HI‐titers than those without ILI, the GMT HI‐titer was low, and almost half of the women had HI < 10, including some with laboratory‐confirmed influenza. Low HI‐titers after PCR‐confirmed pandemic influenza have also been described previously.^33^, ^34^, ^35^ An HI‐titer threshold of 20 has been suggested to provide sufficient serological evidence of infection after a large outbreak with a new strain, as in the 2009 pandemic.^36^ In our study, GMTs below 20 may be explained by mild to moderate illness as reported by 64.9%. Moreover, the median time between influenza exposure and sampling at delivery was longer than the estimated HI‐titer half‐life, causing already low HI‐titers to decrease further. Thus, the timing of the sample collection probably contributed to the low HI‐titers, as demonstrated in another Norwegian study in non‐pregnant patients.^20^

We found no differences in the estimated HI‐titer waning after vaccination with an adjuvanted pandemic vaccine or after mild/moderate ILI. Moreover, the estimated HI‐titer half‐lives indicated that the 39‐day difference in the mean time since exposure could not explain the observed titer difference between these groups. HI‐titer waning appeared slower in women with medically attended, more severe illness; however, there were too few cases to conclude. Studies in non‐pregnant individuals have indicated that waning might be slower after infection than vaccination.^14^, ^15^, ^16^ However, similar to our finding, in the previously mentioned US study of 40 pregnant women, waning was not significantly different after immunization with a non‐adjuvanted vaccine or infection.^17^

Prior to the 2009 pandemic, pregnant women in Norway were not routinely advised to be vaccinated against seasonal influenza and only the pandemic vaccine was recommended for pregnant women during the pandemic.^4^ In the present study, the percentage of self‐reported vaccinations was comparable to the percentage of estimated unregistered pandemic vaccinations nationally.^23^ In addition, the similar HI‐titers against the pandemic virus observed in women who self‐reported pandemic vaccination and in women with a registered pandemic vaccination, further indicate that the women who self‐reported vaccination were indeed vaccinated with the pandemic vaccine.

Ideally, laboratory confirmation should be obtained to establish if ILI is caused by the influenza virus, as opposed to other infectious agents. However, the cohort was population‐based and most women reported moderate illness. Thus, few of the pregnant women were subjected to laboratory testing.^4^, ^37^ This is also in line with a national registry study indicating that most Norwegian pandemic cases during pregnancy were mild to moderate, and among 46 000 pregnant women, only 40 were hospitalized with influenza during the pandemic wave and only 516 cases of laboratory‐confirmed influenza were registered.^4^, ^5^ Since we believe that the real number of pregnant women with pandemic influenza was higher than reflected in the surveillance registry,^4^ we also defined women with a diagnosis of influenza or who self‐reported ILI, as “ILI cases” in our study. By restricting the analyses to ILI occurring during the main pandemic peak, the likelihood that ILI was indeed caused by the pandemic virus was increased.^38^ According to national surveillance data, the pandemic virus was by far the dominating respiratory virus at this time.^38^

As can be expected, medically attended influenza was associated with higher HI‐titers as compared to self‐reported ILI. This could be due to less misclassification of influenza in this group, but may also be the result of more severe illness in the medically attended women, since high HI‐titers were positively associated with severity in these women. Moreover, higher HI‐titers were also observed in women who self‐reported ILI compared to the women with no ILI, implying that they were infected with influenza. Furthermore, according to the questionnaire data, 74.0% of the women with ILI met the CDC definition of ILI, supporting our classification of women without laboratory‐confirmed influenza, as pandemic cases. Some women may nevertheless have been misclassified as infected according to our definition, and some women who experienced pandemic infection outside of the defined pandemic period may have been misclassified as not having ILI.

Information on symptoms was self‐reported in questionnaires listing classical influenza symptoms,^39^, ^40^, ^41^, ^42^ with fever reported as the most common symptom. Typical influenza symptoms (fever, cough, sore throat, shortness of breath, and chest pain) were associated with higher HI‐titers which probably reflects that women reporting these symptoms were indeed influenza cases. Three of these five symptoms are part of the CDC definition of ILI,^27^ and the majority of ILI women fulfilled the CDC definition. Thus, the CDC‐ILI definition may be useful to identify probable ILI cases in this cohort.

A correlation between HI‐titers and disease severity has previously been observed in non‐pregnant patients with A(H1N1)pdm09 infection^20^ and seasonal influenza.^21^ In the latter study, this association was not found for the 2009 pandemic; however, the authors noted that this could be due to too few pandemic cases. In accordance with the positive association between HI‐titers and severity, we have also found an association between T‐cell and NK‐cell responses and ILI symptoms in this pregnancy cohort.^43^ Since data on viral load were lacking, we could not explore if high HI‐titers in the severely ill women were the result of stimulation from a high viral load, and previous studies have been contradictory.^44^, ^45^, ^46^, ^47^, ^48^, ^49^

The strength of this study was that it was conducted within a large, prospective, population‐based pregnancy cohort with both vaccinated and unvaccinated women where an extensive combination of data was available: blood samples collected at delivery, data from several national registries and cohort questionnaire data. The questionnaire data provided valuable information about symptoms and illness. Pregnant women generally pay special attention to their health and as most of the women filled out the questionnaire prior to birth, the reports were not biased by the outcome of the birth.

A limitation of our study was that recruitment of the study participants was not feasible until after the pandemic peak, hence pre‐pandemic or pre‐pregnancy samples could not be obtained and each woman contributed with a single blood sample at delivery. It was therefore not possible to study seroconversion or actual HI‐titer waning in each individual pregnant woman. However, the seroprevalence of antibodies against the pandemic virus was low in the reproductive age groups in Norway prior to the pandemic (HI ≥ 20: 3.9% (30‐49 years)‐10.9% (20‐29 years)),^8^ and we found a similar seroprevalence in the study women with no ILI. A Danish study from the 2009 pandemic found that 17% of pregnant, unvaccinated women were infected based on seroconversion.^30^ This corresponds well with our estimate (19.3%) among unvaccinated women.

To conclude, in this population‐based cohort of women who were pregnant during the 2009 influenza A(H1N1) pandemic, we found that vaccination with the AS03‐adjuvanted pandemic vaccine induced much higher HI‐titers than ILI. 50% of the vaccinated women had HI‐titers ≥20 even 8 months after vaccination. Despite the observed HI‐titer differences between vaccinated women and women with ILI, the estimated HI‐titer waning was similar. Knowledge on the longevity of maternal antibodies is of particular relevance for future influenza pandemics or situations where adjuvanted monovalent vaccines are provided to pregnant women, and may be transferable to seasonal influenza. Moreover, our results indicate that more severe illness may induce higher HI‐titers. Since HI‐titers are influenced by both waning and severity, the generally low HI‐titers measured among the unvaccinated women with ILI may be explained by moderate illness and the long interval in time between exposure and blood sampling.
